# Influencia de las Distintas Variables Clínico-Demográficas en las Características de las Crisis de Migraña

**DOI:** 10.31083/RN44755

**Published:** 2025-12-23

**Authors:** Sonia Quintas, Natalia Aranda Sánchez, Alicia Gonzalez-Martinez, Alba Vieira Campos, Josué Pagán, José Luis Ayala, Javier Galvez-Goicuría, Mónica Sobrado, José Vivancos, Ana Beatriz Gago-Veiga

**Affiliations:** ^1^Servicio de Neurología, Hospital Universitario de La Princesa & Instituto de Investigación Sanitaria La Princesa, 28006 Madrid, España; ^2^Facultad de Medicina, Universidad Autónoma de Madrid, Hospital Universitario de La Princesa, 28029 Madrid, España; ^3^Departamento de Ingeniería Electrónica, Universidad Politécnica de Madrid, 28031 Madrid, España; ^4^Center for Computational Stimulation, Universidad Politécnica de Madrid, Campus de Montegancedo, 28223 Boadilla del Monte, España; ^5^Departamento de Arquitectura de Computadores y Automática, Universidad Complutense de Madrid, 28040 Madrid, España; ^6^Brainguard SL, Pozuelo de Alarcón, 28223 Madrid, España

**Keywords:** migraña, dolor, aplicaciones móviles, factores socio-demográficos, migraña con aura, cognición, migraine, pain, mobile applications, sociodemographic factors, migraine with aura, cognition

## Abstract

**Introducción:**

El diagnóstico de la migraña se basa en criterios clínicos que incluyen diversas características del dolor y síntomas acompañantes, cuya expresión varía entre pacientes. El objetivo de este estudio fue explorar la posible relación entre variables sociodemográficas y las características de las crisis de migraña en un estudio prospectivo en tiempo real.

**Métodos:**

Estudio observacional y longitudinal con recogida prospectiva y en tiempo real de las crisis de migraña mediante una aplicación móvil de diseño propio. Se recopilaron datos clínicos y síntomas acompañantes.

**Resultados:**

Se analizaron 377 crisis en 51 pacientes. Se observó una mayor intensidad de cefalea en mujeres (*p* = 0,038), en pacientes con menor reserva cognitiva (*p* = 0,049) y en aquellos con puntuaciones más altas en la escala Headache Impact Test-6 (HIT-6) (*p* = 0,020). La localización hemicraneal fue más frecuente en pacientes con más años de evolución de la migraña (*p* = 0,028), y ser mujer se asoció a una mayor presencia de náuseas (*p* = 0,034).

**Conclusiones:**

El registro prospectivo y en tiempo real de las crisis de migraña aporta un valor añadido al reflejar de forma más precisa la variabilidad clínica. Los resultados sugieren que factores sociodemográficos influyen en las características de las crisis, apoyando la necesidad de una mayor personalización del tratamiento.

## Abstract (en)

**Introduction:**

Migraine diagnosis relies on clinical criteria that encompass various pain features and associated symptoms, which can vary widely across patients. The aim of this study was to analyze the potential association between sociodemographic factors and the characteristics of migraine attacks.

**Methods:**

We conducted an observational longitudinal study with prospective and real-time data collection using a custom-designed smartphone application. Clinical features and accompanying symptoms of each attack were recorded.

**Results:**

A total of 377 migraine attacks from 51 patients were analyzed. Headache intensity was higher in women (*p* = 0.038), in those with lower cognitive reserve (*p* = 0.049), and in patients with higher Headache Impact Test-6 (HIT-6) scores (*p* = 0.020). Hemicranial pain was more common in patients with a longer disease duration (*p* = 0.028) and female sex was associated with a higher frequency of nausea (*p* = 0.034).

**Conclusions:**

Prospective and real-time recording of migraine attacks provides added value by more accurately capturing their clinical variability. Our findings suggest that sociodemographic variables influence attack characteristics, supporting the need for more personalized therapeutic approaches.

## 1. Introducción

La migraña es una cefalea primaria, no atribuible a alteraciones estructurales cerebrales u otras causas orgánicas, cuyo diagnóstico se basa en criterios clínicos definidos por la *International Classification of Headache Disorders* (ICHD). A lo largo de sus tres ediciones, estos criterios han sido revisados con el objetivo de mejorar su sensibilidad y especificidad diagnóstica [1, 2, 3].

Dichos criterios describen una serie de características del dolor (cualidad pulsátil, localización hemicraneal, intensidad moderada a grave, duración entre 4 y 72 horas, empeoramiento con la actividad física) y síntomas acompañantes (náuseas y/o vómitos, fotofobia, sonofobia), cuya presencia varía entre pacientes. No es necesaria la concurrencia de todos ellos para establecer el diagnóstico de migraña [4].

Además, la crisis de migraña suele comprender varias fases: una fase premonitoria (hasta 48 horas antes del dolor), en la que pueden manifestarse síntomas cognitivos, anímicos, digestivos o vegetativos; seguida del aura (en hasta el 20–25% de los pacientes); la fase de dolor; y finalmente, una fase postdrómica [5, 6].

Esta heterogeneidad clínica sugiere la existencia de mecanismos fisiopatológicos diversos, como las teorías vascular, de sensibilización, trigémino-vascular o glutamatérgica. Factores individuales, como las características sociodemográficas, las comorbilidades o el tiempo de evolución de la enfermedad, podrían influir en estos mecanismos y, por tanto, en la expresión clínica de la migraña [7].

Aunque algunos estudios han intentado explorar estas asociaciones, sus resultados han sido limitados y contradictorios, posiblemente por diseños retrospectivos o transversales, más susceptibles a sesgos, incluidos los de recuerdo [8, 9, 10]. En este contexto, el uso de tecnologías móviles para el registro dinámico y en tiempo real de las crisis representa una herramienta innovadora, con potencial para minimizar estos sesgos y aportar datos más precisos.

El objetivo de nuestro estudio fue describir las características de las crisis de migraña mediante un registro prospectivo y en tiempo real a través de una aplicación móvil específicamente diseñada, y analizar su posible relación con variables sociodemográficas del paciente.

## 2. Material y Métodos

### 2.1 Diseño

Se realizó un estudio observacional y longitudinal en una cohorte de pacientes con migraña episódica. El periodo de inclusión fue de tres meses, durante el cual se efectuó un registro prospectivo y en tiempo real de las crisis mediante una aplicación móvil diseñada específicamente para tal fin. Los pacientes estaban en seguimiento en la Unidad de Cefaleas de un hospital terciario.

Se incluyeron pacientes entre 15 y 69 años con diagnóstico de migraña con o sin aura episódica, realizado por un neurólogo especialista en cefaleas, de acuerdo con los criterios de la tercera edición de la ICHD-III [4], que hubieran sido seguidos durante al menos un año, aceptaran participar voluntariamente en el estudio y firmaran el consentimiento informado. Se excluyeron aquellos con dificultades cognitivas, procesos infecciosos activos o patología aguda que impidieran el correcto registro de las crisis.

### 2.2 Evaluación y Recogida de Datos

Se recogieron variables sociodemográficas, incluyendo: edad, sexo, nivel educativo (primario o universitario), antecedentes familiares, edad de inicio de la migraña, presencia de hipertensión arterial (HTA), diabetes mellitus (DM), dislipemia (DL), índice de masa corporal (IMC), hábito tabáquico, uso de tratamiento preventivo, y puntuaciones en dos escalas: Headache Impact Test-6 (HIT-6), escala de depresión y ansiedad (HAD), y Cognitive Reserve Index Questionnaire (CRIq).

La escala HIT-6 evalúa el impacto de la cefalea sobre la funcionalidad diaria en ámbitos como el trabajo, el hogar, el entorno escolar y las relaciones sociales. Por su parte, la escala de reserva cognitiva incluye ocho ítems organizados en tres dimensiones: (i) educación, (ii) actividad laboral y (iii) tiempo libre. Una mayor puntuación en esta escala se asocia con una mayor reserva cognitiva. El cuestionario de reserva cognitiva se administró al inicio del estudio, fuera de las crisis de migraña, para evitar la influencia de síntomas transitorios sobre las respuestas.

Cada paciente realizó un registro prospectivo y en tiempo real de sus crisis durante un periodo de seguimiento de dos meses. Previamente, recibieron formación sobre el uso de una aplicación móvil desarrollada por el equipo investigador para dicho fin. Se eliminaron los registros duplicados o incompletos. Durante cada crisis, los pacientes consignaron el inicio y fin del dolor, presencia de aura, localización e intensidad del dolor (medida mediante una escala visual analógica [VAS] de 1 a 10), y síntomas acompañantes (náuseas, vómitos, fotofobia, sonofobia, osmofobia).

Para el análisis, las características se agruparon por paciente. Una característica se consideró representativa del tipo de crisis si estaba presente en al menos el 50% de las crisis registradas por ese paciente.

### 2.3 Análisis Estadístico

El análisis se realizó con el software IBM SPSS Statistics, versión 25.0 (IBM Corp., Chicago, IL, USA). Se consideraron estadísticamente significativas las asociaciones con un valor de *p* < 0,05.

La normalidad de las variables se evaluó mediante la prueba de Kolmogorov-Smirnov. Para el análisis de variables cualitativas se utilizó la prueba de chi-cuadrado, aplicando la corrección de Yates cuando las frecuencias esperadas eran menores a 5. La comparación de medias se realizó mediante las pruebas de T de Student o ANOVA, y sus correspondientes versiones no paramétricas (U de Mann–Whitney y Kruskal–Wallis) en función del ajuste a la normalidad. Las correlaciones entre variables cuantitativas se evaluaron mediante los coeficientes de Pearson o rho de Spearman, según correspondiera.

## 3. Resultados

### 3.1 Diagrama de Flujo

**Fig. 1.**
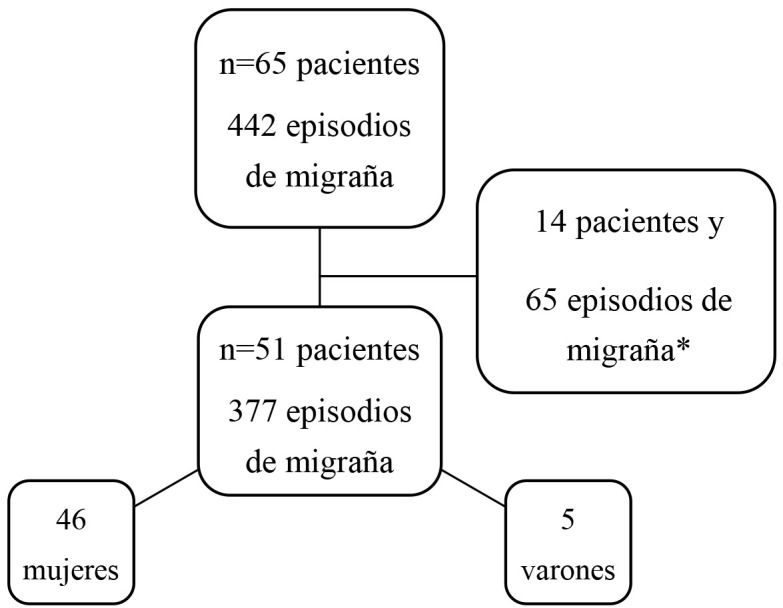
**Diagrama de flujo de la muestra y criterios de inclusión y exclusión**. *Sujetos y episodios de migraña excluidos por presentar datos nulos o repetidos.

En la Fig. 1 se muestra el flujo de inclusión de la muestra. Se analizaron finalmente 51 pacientes (5 varones), con un total de 377 crisis de migraña consideradas válidas para el estudio.

### 3.2 Características Sociodemográficas

Las características basales de los pacientes se detallan en la Tabla 1. De las 377 crisis válidas finalmente analizadas, 316 correspondieron a mujeres y 61 a varones. La mayoría de la muestra correspondió a mujeres (n = 46; 90,2%). La edad media fue de 38,8 ± 10,95 años (38,6 ± 11,19 en mujeres y 40,40 ± 9,3 en varones). Un total de 23 pacientes (45%) presentaban migraña con una evolución de ≥25 años. El 72,5% (37/51) referían antecedentes familiares de migraña, con una prevalencia del 69,6% en mujeres (32/46) y del 100% en varones (5/5). La puntuación media en el cuestionario de reserva cognitiva fue de 16,12 ± 3,3 (16,04 ± 3,2 en mujeres y 16,80 ± 3,9 en varones). La mediana en la escala HIT-6 fue de 63 (Rango Intercuartílico(RIC): 57,5–65,5), sin diferencias relevantes por sexo.

**Tabla 1.** **Características basales de los pacientes incluidos en el estudio**.

	Total muestra	Mujeres (n = 46)	Varones (n = 5)
Edad (años) media ± DE	38,8 ± 10,95	38,6 ± 11,19	40,40 ± 9,30
Edad al diagnóstico (años) media ± DE	14,3 ± 6,20	14,2 ± 6,12	16,20 ± 6,90
% de pacientes con ≥25 años de evolución de la migraña	45,0%	44,4%	60,0%
% de pacientes con antecedentes familiares de migraña	72,5%	69,6%	100,0%
Reserva cognitiva: media ± DE	16,12 ± 3,3	16,04 ± 3,2	16,80 ± 3,9
HIT-6 mediana (intervalo intercuartílico)	63 (57,5–65,5)	63 (58–67)	60 (54–62,5)
% de pacientes con ≥2 #FRV	19,6%	19,6%	20,0%
% de pacientes con HTA	5,9%	6,5%	0,0%
% de pacientes con DM	2,0%	2,2%	0,0%
% de pacientes con DL	11,8%	10,9%	20,0%
% de pacientes fumadores	15,7%	17,4%	0,0%
IMC mediana (intervalo intercuartílico)	23,70 (20,7–27,8)	23,44 (20,5–27,5)	24,40 (23,06–28,2)

DE, Desviación Estándar; #FRV, factores de riesgo cardiovascular (incluyendo HTA, DM, DL, hábito tabáquico e IMC ≥25); HIT-6, Headache Impact Test-6; HTA, hipertensión arterial; DM, diabetes mellitus; DL, dislipemia; IMC, Índice de Masa Corporal.

### 3.3 Aura

Un total de 10 pacientes (19,6%) presentaba criterios diagnósticos de migraña con aura en alguna de sus crisis registradas. Durante el estudio se documentaron 19 crisis con aura del total de 377 crisis analizadas (5,0%). De estas, 9 (17,6%) fueron con aura visual, 6 (11,8%) con aura sensitiva y 4 (7,8%) con aura del lenguaje.

Se observó una asociación significativa entre menores puntuaciones en el cuestionario de reserva cognitiva y la presencia de aura, tanto en el análisis global como para cada subtipo (ver Tabla 2).

**Tabla 2.** **Reserva cognitiva en pacientes en función de la presencia y tipo de aura**.

	Reserva cognitiva media ± DE en pacientes con aura	Reserva cognitiva media ± DE en pacientes sin aura	*p valor*
Aura	13,4 ± 3,5	16,8 ± 2,9	*p* = 0,002**
Aura visual	13,1 ± 3,6	16,8 ± 2,9	*p* = 0,002**
Aura sensitiva	12,8 ± 4,3	16,6 ± 2,9	*p* = 0,008**
Aura del lenguaje	11 ± 4,3	16,6 ± 2,8	*p* = 0,001**

**p < 0,01.

### 3.4 Localización del Inicio del Dolor

La localización más frecuente del inicio de la cefalea fue en una zona concreta, referida por 19 pacientes (37,3%), seguida por la localización holocraneal en 18 (35,3%) y hemicraneal en 9 (17,6%). Un grupo reducido (n = 5; 9,8%) refirió un inicio indistinto entre localización concreta y holocraneal.

**Fig. 2.**
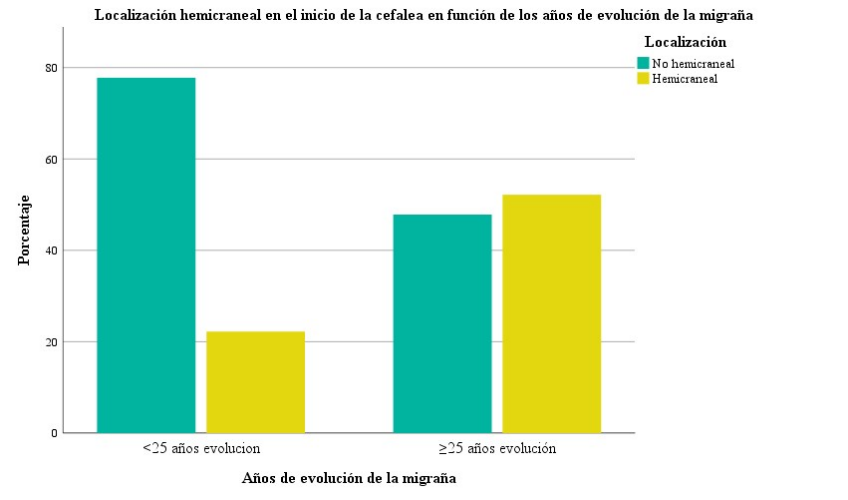
**Gráfico de barras en el que se muestra el porcentaje de pacientes con cefalea de inicio hemicraneal en función de los años de evolución de la migraña**. En el eje de las Y se representa el porcentaje de pacientes que presentan las distintas localizaciones del inicio de la cefalea (hemicraneal o cualquier otra localización), en el eje de las X se representa a los grupos de pacientes en función de los años de evolución de la migraña (menor a 25 años o ≥25 años de evolución).

La cefalea de inicio hemicraneal fue más frecuente en pacientes con una evolución de la migraña ≥25 años (52,2% vs 22,2% en <25 años; *p* = 0,028) (Fig. 2). Cabe señalar que la variable “edad al diagnóstico” se refiere a la edad en que el paciente recibió el diagnóstico clínico de migraña, y que no puede descartarse un posible sesgo por edad, dado que los pacientes de mayor edad tienden a presentar más años de evolución de la enfermedad. No se encontraron otras asociaciones significativas con variables sociodemográficas.

### 3.5 Intensidad del Dolor

Los datos correspondientes se presentan en la Tabla 3. La mediana de la intensidad del dolor fue de 7 (RIC: 6–8) en la muestra global, siendo mayor en mujeres (7 [6–8]) que en varones (6 [4–6,5]); *p* = 0,038.

**Tabla 3.** **Resultados de intensidad de la cefalea en función de las características basales de los pacientes. Los resultados estadísticamente significativos aparecen resaltados**.

Intensidad en relación con:		
Variable sociodemográfica	Resultado test estadístico de contraste^1^	*p* valor
Edad	326,5	*p* = 0,930
Sexo mediana de intensidad (intervalo intercuartílico)	180	*p* = 0,038*
	Mujeres: 7 (6–8)	
	Varones: 6 (4–6,5)	
Edad al diagnóstico de la migraña	–0,081	*p* = 0,575
Años de evolución (≥25 o <25 años de evolución)	334,5	*p* = 0,740
Antecedentes familiares de migraña	232	*p* = 0,559
Reserva cognitiva	–0,280	*p* = 0,049*
HIT-6	0,327	*p* = 0,020*
Presencia de ≥2 #FRV	247	*p* = 0,307
HTA	97	*p* = 0,343
DM	46	*p* = 0,196
DL	118,5	*p* = 0,638
Hábito tabáquico	219	*p* = 0,233
IMC	0,018	*p* = 0,904

^1^Como test estadístico de contraste se aplicó U de Mann-Whitney para todas las variables excepto para edad al diagnóstico de la migraña, reserva cognitiva, HIT-6 e IMC que se aplicó el coeficiente de correlación de Spearman; #FRV, factores de riesgo cardiovascular (incluyendo HTA, DM, DL, hábito tabáquico e IMC ≥25); *p < 0,05.

Una menor reserva cognitiva se asoció a mayor intensidad del dolor (coeficiente de Spearman = –0,280; *p* = 0,049). También se observó una relación positiva entre mayores puntuaciones en HIT-6 y mayor intensidad del dolor (Spearman = 0,327; *p* = 0,020). No se hallaron diferencias significativas con el resto de variables clínicas.

### 3.6 Síntomas Concomitantes

Los síntomas acompañantes más frecuentes fueron la fotofobia (80,4%), sonofobia (58,8%) y náuseas (54,9%). Cabe destacar que ningún varón refirió náuseas, frente al 60,9% de las mujeres (28/46) (*p* = 0,034).

La osmofobia se registró en los tres pacientes con hipertensión arterial (100%), mientras que solo se observó en el 18,8% (9/48) de los pacientes sin hipertensión, diferencia que resultó significativa (*p* = 0,012). No se encontraron otras asociaciones relevantes con variables sociodemográficas.

## 4. Discusión

Nuestro estudio ha analizado la posible relación entre las características basales sociodemográficas de los pacientes con migraña y los síntomas y las características de sus crisis de migraña, de forma prospectiva, a través de una aplicación móvil en tiempo real durante las crisis de dolor.

### 4.1 Diferencias en Función del Sexo

Las mujeres mostraron una mayor intensidad de la cefalea y también una mayor frecuencia de náuseas durante las crisis, sin hallarse diferencias significativas en otros síntomas concomitantes como osmofobia, fotofobia o sonofobia.

Por una parte, la presencia de una mayor intensidad del dolor en mujeres ha sido descrita con anterioridad [8, 9, 11], así como una mayor discapacidad por la enfermedad [8, 12, 13]. El grupo de Scher llevó a cabo un estudio longitudinal en el que participaron 12.495 mujeres y 4294 varones, donde las mujeres presentaron una mayor puntuación en la escala MSSS (Migraine Symptom Severity Scale), que incluye el ítem de dolor moderado-grave [8]. En la revisión realizada por Finocchi y Strada [9] se observó también una mayor intensidad del dolor en mujeres con migraña.

Se ha discutido previamente el papel de las hormonas, los genes y las diferencias en la función y estructura cerebral entre sexos [9, 14]. De esta forma, se ha propuesto la existencia de diferencias estructurales y/o en el procesamiento cerebral del dolor y el grado de activación de distintas áreas en ambos sexos, con una mayor implicación del circuito emocional del dolor en las mujeres [8, 9]. También se ha observado que la enfermedad podría causar una mayor disfunción en la organización de las redes funcionales en reposo en mujeres [13].

En otras enfermedades crónicas que cursan con dolor, como el síndrome del intestino irritable, también se ha descrito un mayor dolor abdominal en mujeres [15]. Diferencias en el procesamiento del dolor, una posible influencia hormonal y factores genéticos se han propuesto en esta entidad como mecanismos implicados en un menor umbral de dolor y una mayor probabilidad de desarrollar enfermedades dolorosas crónicas en mujeres [16, 17].

Existen resultados contradictorios en la literatura científica en cuanto a los síntomas acompañantes, ya que algunos autores refieren distintas proporciones de náuseas, fotofobia, osmofobia y sonofobia entre varones y mujeres [18, 19, 20, 21, 22], mientras que otros estudios no han confirmado esta asociación [23].

Otros aspectos de las crisis de dolor no mostraron diferencias significativas en nuestro estudio, a pesar de que investigaciones previas habían descrito una mayor duración de los episodios en mujeres [13]. También se ha señalado en otros trabajos la necesidad de un mayor tiempo de recuperación tras la crisis, si bien este aspecto no pudo determinarse en nuestro estudio [24].

### 4.2 Diferencias en Función del Tiempo de Evolución de la Migraña

Los pacientes con una cefalea de 25 o más años de evolución presentaron con mayor frecuencia cefalea de inicio hemicraneal. La migraña es una enfermedad crónica con manifestaciones episódicas, por lo que el mayor tiempo con la enfermedad y por tanto de número de crisis experimentadas previamente puede permitir a los pacientes reconocer y definir mejor su dolor a lo largo de los años vividos con la enfermedad.

Estudios previos habrían mostrado una disminución con la edad tanto del impacto de determinados desencadenantes como el estrés, como de aspectos relacionados con el dolor (fotofobia, fonofobia, clinofilia) e incluso del tipo de dolor [25]. También se ha descrito un aumento en la presencia de aura sensitiva y visual con el tiempo de evolución de la enfermedad [26, 27]. Este cambio hacia formas “menos graves” de migraña con la edad se ha relacionado también con aspectos hormonales, pero estos hallazgos no se han confirmado en nuestro estudio.

En cualquier caso, estos datos pueden estar sesgados en nuestro estudio en la medida en que seleccionamos pacientes con migraña episódica, excluyendo aquellos con migraña crónica en los que síntomas como la alodinia intercrítica pueden dificultar el reconocimiento de la topografía del dolor durante las crisis.

### 4.3 Diferencias en Función de la Reserva Cognitiva 

Se encontró una relación entre la reserva cognitiva y la presencia de aura durante las crisis, tanto a nivel general, como para cada uno de los tipos de aura: visual, del lenguaje y sensitiva. Los pacientes con aura presentaban una menor reserva cognitiva que los pacientes sin aura. Este hallazgo podría reflejar una disfunción sutil de redes corticales implicadas tanto en los fenómenos del aura como en procesos cognitivos superiores. Estudios de neuroimagen funcional han mostrado alteraciones de la conectividad y del metabolismo en regiones fronto-insulares, parietales y occipitales en pacientes con migraña con aura, incluso fuera de las crisis. Estas áreas participan en la modulación atencional, la integración sensorial y la autorregulación cognitiva, funciones estrechamente relacionadas con la reserva cognitiva. Así, una menor eficiencia o plasticidad en estas redes podría traducirse en puntuaciones más bajas en los test de reserva cognitiva y, al mismo tiempo, predisponer a la aparición del aura.

Es el primer estudio que muestra esta posible relación, que creemos que ha de ser interpretada con cautela, al ser un cuestionario autoadministrado en el que los pacientes con menor reserva cognitiva podrían haber realizado un falso reconocimiento de otra sintomatología típica acompañante del dolor, como la fatiga mental o los cambios cognitivo-conductuales.

En nuestro estudio también se evidenció una relación inversamente proporcional entre la reserva cognitiva y la intensidad del dolor, encontrándose una mayor intensidad del dolor en pacientes con menor reserva cognitiva. Esto podría explicarse por el hecho de que la reserva cognitiva hace referencia a la capacidad del cerebro de hacer frente al daño que se le presenta a través de estrategias compensadoras o procesos cognitivos presentes previamente; por tanto, los pacientes con una mayor reserva cognitiva podrían tener una mayor capacidad de adaptación al daño que se produce por las distintas patologías [28, 29]. Gomez-Beldarrain *et al*. [30] analizaron mediante resonancia magnética cerebral la integridad de los tractos de sustancia blanca en determinadas regiones cerebrales de interés en pacientes con migraña, entre ellas áreas implicadas en modulación del dolor, cognición y resistencia. De acuerdo con sus resultados, en pacientes con una mayor reserva cognitiva podría existir una mayor actividad en los tractos de sustancia blanca de dichas áreas del cerebro que permiten un mejor control del dolor [30].

### 4.4 Diferencia en Función de la Presencia de Aura

Aunque la comparación de la intensidad del dolor entre pacientes con y sin aura no mostró diferencias significativas, se observó una tendencia a mayor intensidad en los pacientes con aura. Este hallazgo podría sugerir una modulación diferencial de las vías nociceptivas o un mayor grado de sensibilización cortical en este subgrupo, aspectos que deberían confirmarse en futuros estudios con mayor tamaño muestral.

### 4.5 Diferencias en Función del Impacto de la Cefalea

Encontramos una relación directamente proporcional entre la puntuación en la escala HIT-6 y la intensidad de la cefalea, lo que concuerda con la literatura previa donde se señala la intensidad de la cefalea como factor predictor de mayores puntuaciones en dicha escala [31, 32, 33].

### 4.6 Diferencias en Función de las Comorbilidades

En nuestro estudio, no se observaron asociaciones significativas entre las puntuaciones de ansiedad y depresión, evaluadas mediante la escala HADS, y las distintas características analizadas de las crisis de migraña (intensidad, duración, presencia de aura, etc.).

Aunque la ansiedad y la depresión son comorbilidades frecuentes tanto en la migraña episódica como en la crónica, estudios previos han informado resultados heterogéneos al respecto. Algunos trabajos no han encontrado una influencia clara de los síntomas afectivos sobre aspectos clínicos específicos de las crisis de migraña [34], mientras que otros sí han relacionado la patología anímica con una mayor discapacidad y riesgo de cronificación [35]. Estos hallazgos subrayan la necesidad de seguir explorando esta relación en estudios con mayor tamaño muestral y con herramientas específicas para evaluar impacto funcional y calidad de vida.

### 4.7 Limitaciones del Estudio

Este estudio presenta algunas limitaciones que deben tenerse en cuenta al interpretar los resultados. El número de pacientes incluidos fue limitado, aunque se compensa en parte por el elevado número de crisis registradas, lo que proporciona un volumen considerable de datos clínicos. Aunque las correlaciones entre algunas variables alcanzaron significación estadística, su magnitud fue baja. Esta limitada fuerza de asociación podría deberse al tamaño muestral reducido y a la heterogeneidad clínica de la migraña. No obstante, consideramos que estos resultados aportan información relevante como aproximación exploratoria dentro de un diseño prospectivo y con recogida en tiempo real. La escasa representación del sexo masculino también podría haber reducido la capacidad de detectar diferencias estadísticamente significativas en algunas variables analizadas. Asimismo, la exclusión de pacientes con migraña crónica, con el objetivo de caracterizar de manera más precisa los episodios individuales de migraña, puede haber introducido un sesgo de selección, al no representar el espectro completo de la enfermedad. No se realizó un análisis multivariante adicional, ya que el objetivo principal del trabajo fue descriptivo y exploratorio, centrado en identificar posibles tendencias clínicas para estudios posteriores.

Pese a estas limitaciones, una de las principales fortalezas del estudio radica en su diseño prospectivo, con un registro en tiempo real realizado por los propios pacientes, lo que reduce los sesgos de recuerdo típicos de los estudios retrospectivos. Finalmente, no puede descartarse un posible sesgo de atención o efecto Hawthorne [36], ya que el hecho de participar activamente en el estudio podría haber influido en la forma en que los pacientes percibieron e informaron sus síntomas.

## 5. Conclusiones

Los resultados de este estudio sugieren que determinadas variables sociodemográficas, como el sexo, la reserva cognitiva y el impacto funcional de la cefalea (evaluado mediante HIT-6), se asocian con características clínicas específicas de las crisis de migraña, incluyendo la intensidad del dolor, la presencia de aura y de síntomas acompañantes como las náuseas. En particular, las mujeres presentaron mayor intensidad del dolor y mayor frecuencia de náuseas, mientras que una menor reserva cognitiva se relacionó con mayor intensidad del dolor y con la presencia de aura. La recogida en tiempo real de las crisis mediante aplicación móvil refuerza la validez de los hallazgos y reduce posibles sesgos de recuerdo.

Estos hallazgos refuerzan la hipótesis de que la expresión clínica de la migraña está modulada, al menos en parte, por factores individuales, y destacan la importancia de considerar dichas variables en práctica clínica. Estudios futuros con un mayor tamaño muestral podrían reforzar estas asociaciones y contribuir al desarrollo de estrategias de tratamiento más personalizadas, orientadas a mejorar la calidad de vida de los pacientes con migraña.

## Data Availability

Los datos presentados en este estudio están disponibles previa solicitud al autor de correspondencia, debido a restricciones éticas y legales relacionadas con la protección de información clínica sensible. Los datos fueron anonimizados y almacenados en una base de datos para su posterior análisis estadístico. Estos datos solo podrán compartirse con profesionales sanitarios cualificados e investigadores especializados en el ámbito de la cefalea que acepten cumplir las condiciones institucionales de uso de datos y justifiquen adecuadamente su necesidad.

## Contribuciones de los Autores

ABGV, JV, SQ, AVC, MS, JLA y JP diseñaron el estudio. SQ, NAS, AGM y ABGV redactaron el manuscrito. JGG, JP y JLA realizaron el análisis de los datos. AGM, SQ, NAS, MS, AVC y ABGV llevaron a cabo la recogida de datos. ABGV, MS y JV revisaron el manuscrito. Todos los autores contribuyeron a los cambios editoriales en el manuscrito. Todos los autores leyeron y aprobaron la versión final del manuscrito. Todos los autores han participado suficientemente en el trabajo y han aceptado ser responsables de todos los aspectos del mismo.

## Aprobación Ética y Consentimiento Informado

El estudio fue aprobado por el comité de ética del Hospital Universitario de la Princesa (número de registro 3919). Todos los pacientes firmaron el consentimiento informado por escrito. El estudio se realizó conforme a los principios de la Declaración de Helsinki.

## Financiación

Este trabajo ha sido financiado por el Instituto de Salud Carlos III (ISCIII) y Fondo Europeo de Desarrollo Regional (FEDER), a través del proyecto IMPaCT PMP22/00158, y cofinanciado por la Unión Europea mediante el Plan de Recuperación, Transformación y Resiliencia – Next Generation EU, otorgado a SQ, AGM, JV y ABGV.
